# Hard on the Regulars

**Published:** 1890-05

**Authors:** 


HARD ON THE REGULARS.—A regular physician was summoned to attend a member of Mrs Malaprop's family; he expressed some surprise at being called, knowing the lady in question to be an ardent follower of homœopathy. "Yes," said Mrs. M., "I have changed my views, the homœopaths are good for infant-ry, but the regulars are best for adult-ry.—*Med. World.*

